# Hydrogen Sulfide Alleviates Aluminum Toxicity via Decreasing Apoplast and Symplast Al Contents in Rice

**DOI:** 10.3389/fpls.2018.00294

**Published:** 2018-03-06

**Authors:** Chun Q. Zhu, Jun H. Zhang, Li M. Sun, Lian F. Zhu, Buhailiqem Abliz, Wen J. Hu, Chu Zhong, Zhi G. Bai, Hussain Sajid, Xiao C. Cao, Qian Y. Jin

**Affiliations:** ^1^State Key Laboratory of Rice Biology, China National Rice Research Institute, Hangzhou, China; ^2^State Key Laboratory of Soil and Sustainable Agriculture, China Institute of Soil Science, Chinese Academy of Sciences, Nanjing, China; ^3^Nuclear Technology Biotechnology Research Institute, Xinjiang Academy of Agricultural Sciences, Ürümqi, China; ^4^Zhejiang Academy of Agricultural Sciences, Hangzhou, China

**Keywords:** aluminum toxicity, hydrogen sulfide, cell wall, root elongation, gene expression, rice, antioxidant enzymes

## Abstract

Hydrogen sulfide (H_2_S) plays a vital role in Al^3+^ stress resistance in plants, but the underlying mechanism is unclear. In the present study, pretreatment with 2 μM of the H_2_S donor NaHS significantly alleviated the inhibition of root elongation caused by Al toxicity in rice roots, which was accompanied by a decrease in Al contents in root tips under 50 μM Al^3+^ treatment. NaHS pretreatment decreased the negative charge in cell walls by reducing the activity of pectin methylesterase and decreasing the pectin and hemicellulose contents in rice roots. This treatment also masked Al-binding sites in the cell wall by upregulating the expression of *OsSATR1* and *OsSTAR2* in roots and reduced Al binding in the cell wall by stimulating the expression of the citrate acid exudation gene *OsFRDL4* and increasing the secretion of citrate acid. In addition, NaHS pretreatment decreased the symplasmic Al content by downregulating the expression of *OsNRAT1*, and increasing the translocation of cytoplasmic Al to the vacuole via upregulating the expression of *OsALS1*. The increment of antioxidant enzyme [superoxide dismutase (SOD), ascorbate peroxidase (APX), catalase (CAT), and peroxidase (POD)] activity with NaHS pretreatment significantly decreased the MDA and H_2_O_2_ content in rice roots, thereby reducing the damage of Al^3+^ toxicity on membrane integrity in rice. H_2_S exhibits crosstalk with nitric oxide (NO) in response to Al toxicity, and through reducing NO content in root tips to alleviate Al toxicity. Together, this study establishes that H_2_S alleviates Al toxicity by decreasing the Al content in the apoplast and symplast of rice roots.

## Introduction

Al toxicity is one of the most widespread factors limiting crop production, especially in acid soils. Approximately 30–50% of arable lands worldwide (and ∼21% in China) are acidic (He et al., 2012). The most important symptom of Al toxicity is the inhibition of root growth. Once Al toxicity disturbs roots, the uptake of water and nutrient elements is impaired, thereby constraining crop growth and yields (Blamey et al., 2004; Ma, 2005). The formation of an excess of reactive oxygen species (ROS) such as H_2_O_2_ and O_2_^-^ in plant cells is another primary response to Al toxicity, as detected in soybean root tips (Horst et al., 1992), *Cassia tora* L. (Wang and Yang, 2005), Melaleuca trees (Tahara et al., 2008), rice leaves (Kuo and Kao, 2003), and cultured tobacco (*Nicotiana tabacum* L.) cells (Devi et al., 2003). The increased ROS levels severely damage the integrity of lipid membranes and limit plant growth. Other phytotoxic effects of Al have also been observed, such as disrupted Ca^2+^ homeostasis in the cytoplasm (Rengel, 2004), callose deposits at the plasmodesmata in roots (Sivaguru et al., 2000), and damaged respiration in the mitochondria (Yamamoto et al., 2002).

Plants have developed various strategies to cope with Al toxicity, including detoxifying Al inside the cell and inhibiting the deposition of Al in the apoplast or reducing Al transfer from the outside environment to the cell. Increasing the activities of the antioxidant enzymes superoxide dismutase (SOD), ascorbate peroxidase (APX), guaiacol peroxidase (GPX), and catalase (CAT) to alleviate damage from ROS is a common way in which Al toxicity is alleviated in plants (Wang and Yang, 2005; Zhang et al., 2010b). Wang et al. (2017) recently found that the activity of cytosolic glucose-6-phosphate dehydrogenase is also involved in resistance to Al through mediating ROS levels in soybean (Wang et al., 2017). In addition, plants alleviate Al toxicity by combining Al with organic compounds to detoxify it in the cytoplasm, for example, combining Al with citrate in buckwheat or with delphinidin-3-glucoside and 3-caffeolylquinic acid in *Hydrangea macrophylla* (Ma et al., 1998; Ma and Hiradate, 2000; Schreiber et al., 2011). Excluding Al via the exudation of carboxylates from roots is a widespread mechanism found in plants that are resistant to Al toxicity, including the exudation of citrate in rice, maize, and soybean (Silva et al., 2001; Ma et al., 2002; Pineros et al., 2002), of malate in wheat and radish (Zheng et al., 1998a; Papernik et al., 2001), and of oxalate in taro and buckwheat (Ma et al., 1997; Ma and Miyasaka, 1998). Compartmentalizing Al in the vacuole is another important way to detoxify Al inside the plant (Shen et al., 2002). Finally, increasing the pH in the rhizosphere efficiently alleviates Al toxicity in Arabidopsis (Degenhardt et al., 1998).

The cell wall is the first cellular component that directly encounters Al. Most Al is fixed in the cell walls of plant. For example, approximately 85–90% and 99.9% of total Al is bound in the root cell wall of barley and in the cell wall of the giant alga *Chara corallina*, respectively (Clarkson, 1967; Rengel and Reid, 1997). Decreasing cell wall Al content through modifying the cell wall structure and polysaccharide content can significantly alleviate Al toxicity. Wang et al. (2015) demonstrated that the application of NH_4_^+^ decreased the Al^3+^ content in rice roots through decreasing the pectin content in rice roots (Wang et al., 2015). The degree of pectin methylation and the pectin content in roots determine the capacity for Al resistance in rice, and the hemicellulose content in Arabidopsis roots similarly determines its Al resistance because the Al is mainly deposited in hemicellulose in Arabidopsis (Yang et al., 2008, 2011). Thus, reducing the cell wall Al content is an efficient strategy to decrease Al toxicity in plants.

Hydrogen sulfide (H_2_S) has long been considered a phytotoxin due to its toxic effects on plant growth and development (García-Mata and Lamattina, 2013). For example, exposing plants to high H_2_S levels damages their leaves and inhibits plant growth (Nakamura et al., 2009). However, in recent years, the role of H_2_S has been re-evaluated because of its positive effect on plant growth and its mediation of abiotic and biotic stress resistance in plants at low concentrations. In low doses, H_2_S functions as a signaling molecule in various physiological processes, such as improving wheat seed germination under osmotic stress (Zhang et al., 2010c), regulating the opening and closing of stomata in Arabidopsis (Liu et al., 2011; Jin et al., 2013), and improving resistance to salt stress in alfalfa (Wang et al., 2012), heavy metals such as Al in barley (Chen et al., 2013), and oxidative stress in wheat under water-stress conditions (Shan et al., 2011). However, the role of H_2_S in alleviating Al toxicity in rice and the underlying mechanism remain elusive.

The rice cultivar Nipponbare was selected in the current study due to its high tolerance to Al. The rice seedlings were pretreated with the H_2_S donor NaHS prior to exposure to Al to study the effects of H_2_S on the alleviation of Al toxicity. The results of this study indicate that H_2_S alleviates Al toxicity via reducing the cell wall and symplast Al contents when rice is exposed to Al.

## Materials and Methods

The *japonica* rice Nipponbare seeds were sterilized in 1% (*v*/*v*) sodium hypochlorite solution (NaClO). After 10 min, the seeds were washed three times with deionized water, followed by soaking in deionized water. After 1 day, the seeds were germinated on moist filter paper for 24 h. The germinated seeds were transferred to a net floating on 0.5 mM CaCl_2_ (pH 5.5) solution in a 5-liter plastic container. When the roots were approximately 1 cm long, various concentrations of NaHS (to a final concentration of 0, 1, 2, 5, and 10 μM) were added to fresh CaCl_2_ (pH 4.5) solution. After 8 h, the CaCl_2_ solution was discarded and the seedlings were separated into two groups: one group was transferred to fresh 0.5 mM CaCl_2_ (pH 4.5), and the other group was transferred to 0.5 mM CaCl_2_ (pH 4.5) containing different concentrations (30, 50, and 100 μM) of AlCl_3_. Root length was measured before and after Al treatment with a ruler. All experiments were performed in an environmentally controlled growth room at 30°C and a relative humidity of 60%.

For the NO and hypotaurine addition experiment, eight different treatments were performed as follows: CK, +Al, +Al+NaHS, +Al+HP, +Al+SNP, and +Al+c-PTIO. The final concentrations of the treatments were 2 μM NaHS, 100 μM hypotaurine (HP), 2.5 μM sodium nitroprusside (SNP), 10 μM 2-(4-carboxyphenyl)-4,4,5,5-tetramethylimidazoline-1-oxyl-3-oxide (c-PTIO), and 50 μM Al. NaHS and SNP were added to the CaCl_2_ solution, and 8 h later, the seedlings were transferred to 0.5 μM AlCl_3_ solution; HP and c-PTIO were applied together with 0.5 μM AlCl_3_. The pH of the solution was adjusted to 4.5.

### Extraction of the Root Cell Wall and Its Components

Fresh rice roots were ground with liquid nitrogen, followed by extraction with (in order) 75% alcohol, acetone, a mixture of methyl alcohol and chloroform (1:1, *v*/*v*), and methyl alcohol. The resulting crude cell wall fraction was freeze dried, and pectin was extracted from the cell walls three times with 1 mL of distilled water at 100°C. The resulting pellet (CW-pectin) was subsequently extracted twice by incubation in 24% KOH (containing 0.02% KBH_4_) for 12 h each time. The supernatant was collected after centrifugation at 16,000 × *g* for 5 min and pooled to yield the hemicellulose fraction (Zhong and Lauchli, 1993; Yang et al., 2011; Zhu et al., 2012b).

### Measurement of Cell Wall Components

The pectin content was measured based on uronic acid content, and the hemicellulose content was measured based on total sugar content, as described previously (Dubois et al., 1956; Blumenkrantz and Asboe-Hansen, 1973).

### Determination of Al Contents in Different Fractions

#### Al Content in Root Tips

The root tips (0–1 cm) were collected with a razor blade, and samples comprising 10 root tips were soaked in 1 mL of 2 M HCl solution to extract Al. After 24 h, the supernatant was collected and diluted with 3 mL of ultrapure water, and the Al content was measured by inductively coupled plasma mass spectrometry (ICP-MS).

#### Al Content in Cell Sap

The root tips (0–1 cm) were collected with a razor blade, and samples comprising 10 root tips were placed on a centrifugal filter (0.45 μM) and frozen at 80°C overnight. Then, the frozen samples in the centrifugal filters were thawed at 25°C to release cell sap. After centrifugation at 20,600 × *g* for 10 min, the volume of cell sap was recorded, and the sap was diluted with 2 mL of ultrapure water (Xia et al., 2010). The Al content was measured by ICP-MS.

#### Al Content in Cell Walls

Approximately 2 mg of cell wall material was mixed with 1 mL of HCl (2 M) and placed in a vortex vibration meter. After 1 day, the supernatant was collected by centrifugation at 20,600 × *g* for 10 min and diluted in 2 mL of ultrapure water. The Al content was measured by ICP-MS.

### Eriochrome Cyanine R Staining

Roots were washed in 0.5 mM CaCl_2_ three times and stained with 5 mL of 0.1% eriochrome cyanine R (*v*/*w*) for 10 min. The stained roots were washed with distilled water and observed with a MicroPublisher 3.3 RTV equipped with a digital microscope camera (QImaging, Canada). The pink color on the root surface indicates Al accumulation.

### Pectin Methylesterase Activity Assay

The enzyme pectin methylesterase (PME) was extracted with 10 mM Tris buffer (pH 7.7) containing 1 mol/L NaCl. After 20 min incubation on ice, the samples were centrifuged at 18,500 × *g* at 4°C for 10 min, and the supernatant was collected and used to measure PME activity as described previously (Anthon and Barrett, 2004; Zhu et al., 2012a).

### Measurement of Citrate Acid Efflux From Roots

The root exudates from plants under various treatments were collected using a cation and anion exchange column successively according to Zheng et al. (1998b). Fresh roots were weighed, and the citrate acid content in roots was measured via ion chromatography after the eluent was concentrated in a rotary evaporator at 40°C (Zhu et al., 2014).

### Measurement of Enzyme Activities

Fresh rice roots were collected and weighed, then ground with 2 mL of extraction buffer (50 mM PBS, pH7.8, 1 mM EDTA-Na_2_ and 1 mM ascorbic acid) in an ice bath. The supernatant was regarded as the crude enzyme solution and collected by centrifugation at 15,000 × *g* for 15 min at 4°C.

The activity of SOD was detected by determining its ability to inhibit nitroblue tetrazolium (NBT) photochemical reduction. A 3-mL reaction mixture containing 1.5 mL 50 mM PBS (pH7.8), 0.3 mL 20 μM riboflavin, 0.3 mL 150 mM L-methionine, 0.3 mL 750 μM NBT, 0.3 mL 100 μM EDTA-Na_2_, 0.25 mL H_2_O, and 50 μL crude enzyme solution was incubated for 20 min under a white fluorescent lamp. A reaction without enzymes was used as a negative control. The absorbance of the solution was measured at 560 nm (Beauchamp and Fridovich, 1971).

Catalase activity was assayed based on the decrease of H_2_O_2_ content. A reaction mixture containing 1.9 mL 50 mM PBS (pH 7.0), 1 mL 0.1% H_2_O_2_ and 0.1 mL crude enzyme solution was prepared. The absorbance value at 240 nm was recorded at the beginning and 1 min after the reaction began (Dhindsa et al., 1981).

Ascorbate peroxidase activity was measured according to the decrease of ascorbate content. The 3-mL reaction mixture contained 1.5 mL 50 mM potassium phosphate buffer (pH 7.0), 0.5 mL 0.3 mM sodium ascorbate, 0.5 mL 0.1 mM EDTA-Na_2_, 0.4 mL 0.06 mM H_2_O_2_, and 0.1 mL of crude enzyme solution. The absorbance was recorded at 290 nm at 10 s and 30 s after the reaction began (Nakano and Asada, 1981).

Peroxidase activity was assayed according to the tetraguaiacol production rate and the 3-mL reaction mixture contained 1 mL 50 mM potassium phosphate buffer (pH 5.5), 0.95 mL 0.2% guaiacol, 1 mL 0.3% H_2_O_2,_ and 50 μL of crude enzyme solution. The absorbance was recorded at 470 nm at 30 s and 90 s after the reaction began (Chen et al., 2013).

### Measurement of Lipid Peroxidation

Lipid peroxidation was estimated via determining the content of malondialdehyde (MDA) according to Wang et al. (2017). Fresh rice roots (about 0.2 g) were ground and extracted in 2 mL of 10% (*w*/*v*) trichloroacetic acid; then the supernatant was collected by centrifuging at 8,000 × *g* for 10 min at 4°C. A 1.5-mL aliquot of the supernatant was used to determine the MDA content by addition of 1.5 mL reaction mixture (containing 0.5% thiobarbituric acid and 10% trichloroacetic acid) and incubation at 95°C for 30 min. After cooling on an ice bath, the reaction mixtures were centrifuged at 10,000 × *g* for 10 min at 25°C, and the supernatant were collected and measured at 440, 532, and 600 nm, respectively.

### Assay of H_2_O_2_ Content

The fresh rice roots (about 0.2 g) were collected and ground with 2 mL of cooled acetone. Then, the supernatants were collected after centrifugation at 8,000 × *g* for 10 min at 4°C. Next, 0.1 mL of concentrated ammonia and 0.1 mL of 5% TiSO_4_ were applied to 1 mL supernatant and mixed. The reaction mixture was centrifuged at 4,000 × *g* for 10 min at 25°C and the supernatant discarded. The residue was dissolved in 4 mL of 2 M H_2_SO_4_. After 5 min of incubation at 25°C, the absorbance was recorded at 415 nm (Wang et al., 2017).

### Determination of NO Contents in Roots

Root tips (0–1 cm) were collected and washed with 20 mM HEPES-KOH (pH 7.4) for 15 min at room temperature. Then, the collected roots were incubated with 0.5 mL of 4-amino-5-methylamino-2,7-difluorofluorescein diacetate (DAF-FM DA) probe (10 μM) in complete darkness. After 30 min, about 10 mL of 20 mM HEPES-KOH (pH 7.4) was used to wash the stained root tips three times, for 20 min each time. Micrographs were obtained with a Nikon Eclipse 80i fluorescence microscope, Nikon, EX 460–500, DM 505, BA 510–560. The intensity of fluorescence was calculated using PhotoShop 7.0 software (Adobe Systems) (Besson-Bard et al., 2009).

### Assay of H_2_S Content

Hydrogen sulfide and dimethyl-*p*-phenylenediamine in H_2_SO_4_ can form methylene blue, which absorbance can be measured at 667 nm (Li et al., 2013). Fresh rice roots (0.5 g) were ground in 2 mL phosphate buffer (pH 6.8, 50 mM) containing 200 mM ascorbic acid and 0.1 mM EDTA-Na_2_. The supernatant was collected by centrifugation at 20,600 × *g* for 10 min and transferred into a test tube. Next, 2 mL of reaction solution containing 100 mM PBS (pH 7.4), 2 mM phosphopyridoxal and 10 mM L-cysteine, and 0.2 mL of zinc acetate (0.1%, *w*/*v*) was added into the tube. The mixture solution was incubated at room temperature for 30 min, and 0.15 mL of 5 mM dimethyl-*p*-phenylenediamine (dissolved in 3.5 mM H_2_SO_4_) was added to the tube, followed by 0.15 mL of 50 mM ferric ammonium sulfate (dissolved in 100 mM H_2_SO_4_). After 15 min at 25°C, the absorbance of the solution was monitored at 667 nm. A reaction without zinc acetate addition served as a blank.

### Gene Expression Analysis

Rice roots were harvested, immediately frozen in liquid nitrogen, and ground for total RNA extraction. Total RNA extraction, reverse transcription, and PCR were performed according to Zhu et al. (2016). The sequences of the gene-specific primers are shown in **Table 1** (Xia et al., 2010, 2013; Kengo et al., 2011; Huang et al., 2012). Each cDNA sample was run in triplicate. Relative gene expression levels were calculated via the 2^-ΔΔ*C*_T_^ method.

**Table 1:** Primers used in the present study.

Gene	Sequence (5′–3′)
*OsFRDL4*-F	CGTCATCAGCACCATCCACAG
*OsFRDL4*-R	TCATTTGCGAAGAAACTTCCACG
*OsSTAR1*-F	TCGCATTGGCTCGCACCCT
*OsSTAR1*-R	TCGTCTTCTTCAGCCGCACGAT
*OsSTAR2*-F	ACCTCTTCATGGTCACCGTCG
*OsSTAR2*-R	CCTCAGCTTCTTCATCGTCACC
*OsNRAT1*-F	GAGGCCGTCTGCAGGAGAGG
*OsNRAT1*-R	GGAAGTATCTGCAAGCAGCTCTGATGC
*OsALS1*-F	GGTCGTCAGTCTCTGCCTTCTC
*OsALS1*-R	CCTCCCCATCATTTTCATTTGT
*OsHistone*-F	GGTCAACTTGTTGATTCCCCTCT
*OsHistone*-R	AACCGCAAAATCCAAAGAACG


### Statistical Analysis

Data were analyzed by one-way ANOVA, and the mean values were compared by Tukey’s post-test using SPSS (version 21.0.0; IBM, Armonk, NY, United States). Different letters on the graphs indicate that the mean values were statistically different at the *P* < 0.05 level.

## Results

### Pretreatment With NaHS Significantly Alleviates Al Toxicity

**FIGURE 1:**
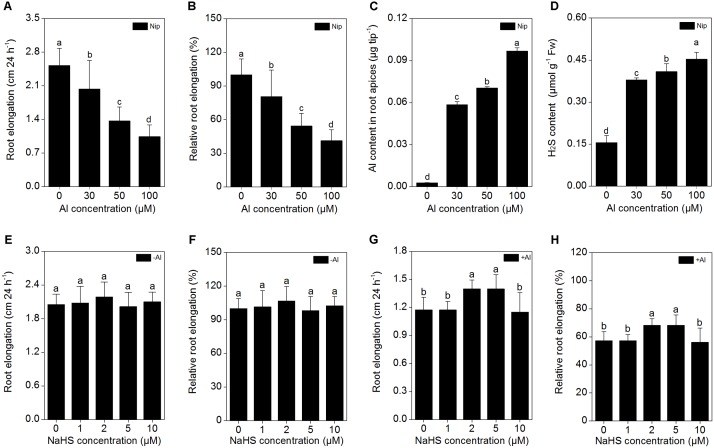
Effects of different Al^3+^ concentrations on root elongation **(A)**, relative root elongation **(B)**, Al content in root apices **(C)**, H_2_S content in root **(D)**, and effects of different NaHS concentrations on root elongation **(E)**, relative root elongation **(F)** under Al-absent conditions and root elongation **(G)**, relative root elongation **(H)** under 50 μM Al^3+^ conditions. Data are means ± SD (*n* = 10). Columns with different letters are significantly different at *P* < 0.05.

The *japonica* rice variety Nipponbare was used in the present study to gain insight into the role of H_2_S in Al resistance. Seedlings with roots of approximately 3 cm in length were treated with 0, 30, 50, and 100 μM Al^3+^ in 0.5 M CaCl_2_ (pH 4.5) solution for 24 h, then root length, Al^3+^ content in root apices and root H_2_S content were measured. As shown in **Figures 1A–C**, there was a significant decrease in root length and an increase of Al^3+^ content in root tips with increasing Al^3+^ level in the solution. Root length was reduced by approximately 50% in the presence of 50 μM Al^3+^ vs. the control, and therefore, 50 μM Al^3+^ was used in subsequent experiments. With increasing Al^3+^ concentrations in the solution, the H_2_S content in the rice roots also significantly increased (**Figure 1D**), indicating that H_2_S may be involved in the rice response to Al toxicity.

**FIGURE 2:**
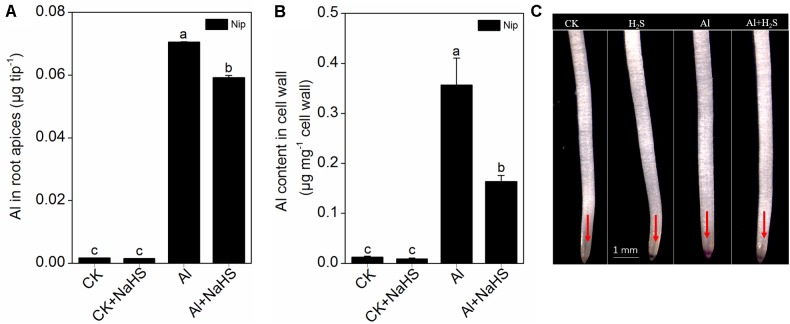
Effect of 2 μM NaHS on Al contents in root apices **(A)**, root cell wall **(B)** and the Al deposited at the root surface **(C)**. The root tips (0–1 cm) were stained with 0.1% eriochrome cyanine R (v/w) and the pink color on the root surface indicates Al accumulation (as the red arrows point out). Data are means ± SD (*n* = 4). Columns with different letters are significantly different at *P* < 0.05.

Next, the rice seedlings were pretreated with different concentrations (0, 1, 2, 5, 10 μM) of NaHS. After 8 h, the seedlings were transferred to 0.5 mM CaCl_2_ (pH 4.5) solution with or without 50 μM Al^3+^, and root length and Al^3+^ content in the root tips were measured 24 h later. As shown in **Figures 1E,F**, NaHS had no significant effect on root length under Al^3+^-absent conditions. However, root length significantly increased in seedlings pretreated with 2 and 5 μM NaHS under 50 μM Al^3+^ treatment (**Figures 1G,H**), indicating that NaHS significantly alleviated the inhibition of root growth under Al^3+^ conditions. The 2 μM NaHS concentration was selected for further analysis; the Al^3+^ content in root tips significantly decreased under 2 μM NaHS pretreatment (**Figure 2A**), indicating that the H_2_S decreased the Al content in rice roots, thereby alleviating the inhibited root growth induced by Al toxicity.

### H_2_S Reduces the Al Content in Rice Roots by Modifying Cell Wall Components and Increasing Citrate Secretion

**FIGURE 3:**
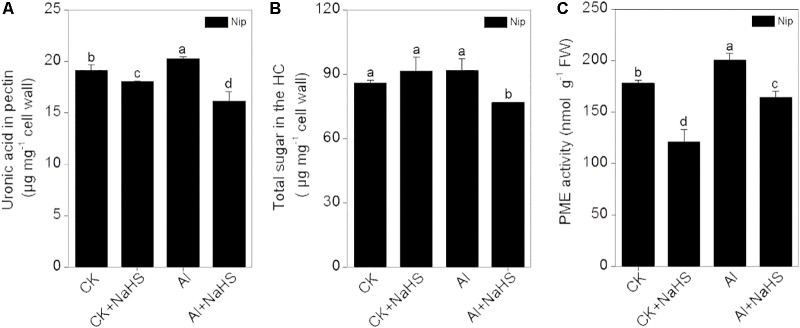
Effect of 2 μM NaHS on pectin content **(A)** and hemicellulose content **(B)** in the root cell wall and the activity of pectin methylesterase (PME) in rice roots **(C)**. Data are means ± SD (*n* = 4). Columns with different letters are significantly different at *P* < 0.05.

**FIGURE 4:**
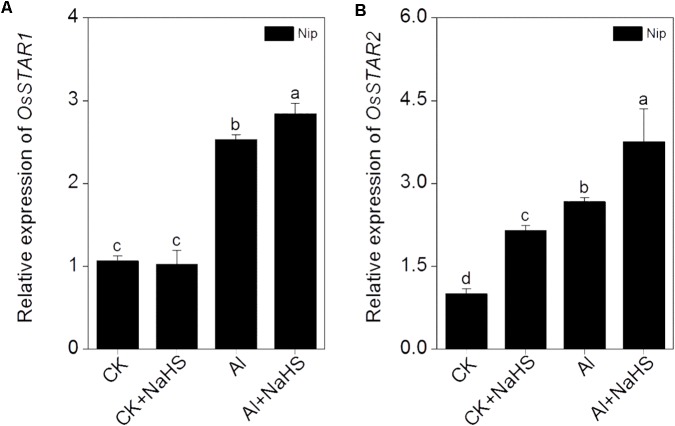
Effect of 2 μM NaHS on the expression of *OsSTAR1* **(A)** and *OsSTAR2* **(B)**. Data are means ± SD (*n* = 4). Columns with different letters are significantly different at *P* < 0.05.

Most Al in the root is contained in the cell wall. Under high Al conditions, Al can be released from the cell wall through a change in the degree of methylation of pectin in the cell wall and in the content of hemicellulose, thereby alleviating Al toxicity (Clarkson, 1967; Yang et al., 2008, 2011). Therefore, the rice root cell walls and the components in the cell wall were extracted and analyzed in the present study. As shown in **Figure 2B**, pretreatment with 2 μM of the H_2_S donor NaHS significantly reduced the Al content in the cell walls of rice roots compared to the control under 50 μM Al^3+^ conditions. To visualize Al deposits at the root surface, the root segments (0–1 cm) were further stained with eriochrome cyanine R, demonstrating that the Al levels (stained pink) were significantly decreased by pretreatment with NaHS under 50 μM Al^3+^ conditions (**Figure 2C**), indicating that H_2_S decreased the binding of Al in the root surface. As pectin and hemicellulose are the two major polysaccharides in rice root cell walls that contribute to the release of Al from this structure (Yang et al., 2008, 2011), their contents in rice root cell walls were also measured. As shown in **Figure 3**, the pectin and hemicellulose contents in root cell walls both decreased in response to 2 μM NaHS pretreatment in the presence of 50 μM Al^3+^, which was accompanied by a decrease in PME activity in roots, suggesting that H_2_S treatment may alleviate Al toxicity in rice roots under high-Al conditions by decreasing the contents of cell wall polysaccharose and altering its structure. In addition, the expressions of *OsSTAR1* and *OsSTAR2*, which modify the cell wall to reduce cell wall Al contents (Delhaize et al., 2012; Huang et al., 2012), were also significantly increased in the presence of NaHS under high-Al conditions (**Figure 4**).

**FIGURE 5:**
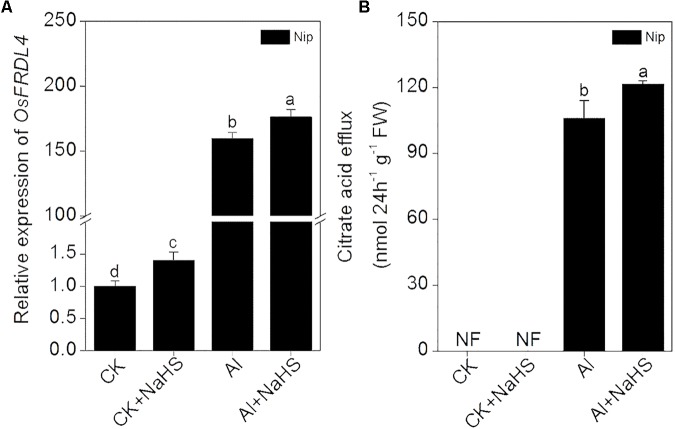
Effect of 2 μM NaHS on the expression of *OsFRDL4* in rice roots **(A)** and the secretion of citrate acid from rice roots **(B)**. Data are means ± SD (*n* = 4). Columns with different letters are significantly different at *P* < 0.05.

Increasing the secretion of organic acids from the root to decrease Al deposition at the root surface is another efficient mechanism used to alleviate Al toxicity (Ma et al., 2001), and OsFRDL4 is responsible for the efflux of citrate acid from rice roots (Yokosho et al., 2011). The expression of *OsFRDL4* was significantly increased in response to NaHS pretreatment under 50 μM Al^3+^ conditions, accompanied with a significant increase in the secretion of citrate from roots (**Figure 5**), indicating that H_2_S also increases the secretion of citrate from the roots to decrease Al deposition, thereby alleviating Al toxicity.

### H_2_S Reduces Al^3+^ Transport to the Cell Interior and Increases Translocation of Al to the Vacuole

**FIGURE 6:**
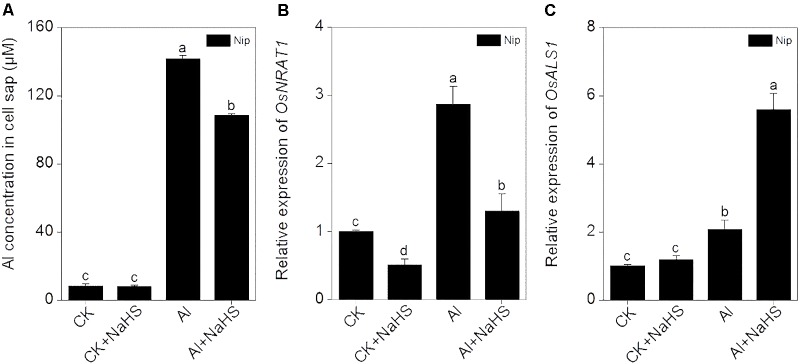
Effect of 2 μM NaHS on Al concentration in cell sap **(A)** and the expression of *OsNRAT1* **(B)** and *OsALS1* **(C)** in rice roots. Data are means ± SD (*n* = 4). Columns with different letters are significantly different at *P* < 0.05.

OsNRAT1 is responsible for the transport of Al^3+^ from the environment to the interior of the cell (Xia et al., 2010). After pretreatment with NaHS, the expression of *OsNRAT1* significantly decreased in roots, which was accompanied by a decrease in Al levels in cell sap under Al toxicity conditions (**Figure 6**), suggesting that H_2_S reduces the transfer of Al^3+^ from the outside environment to the inside of the cell to alleviate its inhibitory effect on root elongation. Once Al enters the plant cell, it must be detoxified. OsALS1 is involved in translocating Al from the cytoplasm to the vacuole, thereby alleviating Al toxicity in plants (Huang et al., 2012). In the current work, the expression of *OsALS1* in rice roots significantly increased in response to H_2_S treatment under 50 μM Al^3+^ conditions (**Figure 6**), indicating that H_2_S stimulates the transport of Al to the vacuole via OsALS1.

### H_2_S Stimulates the Activity of Antioxidant Enzymes to Reduce ROS Damage in Rice Roots

**FIGURE 7:**
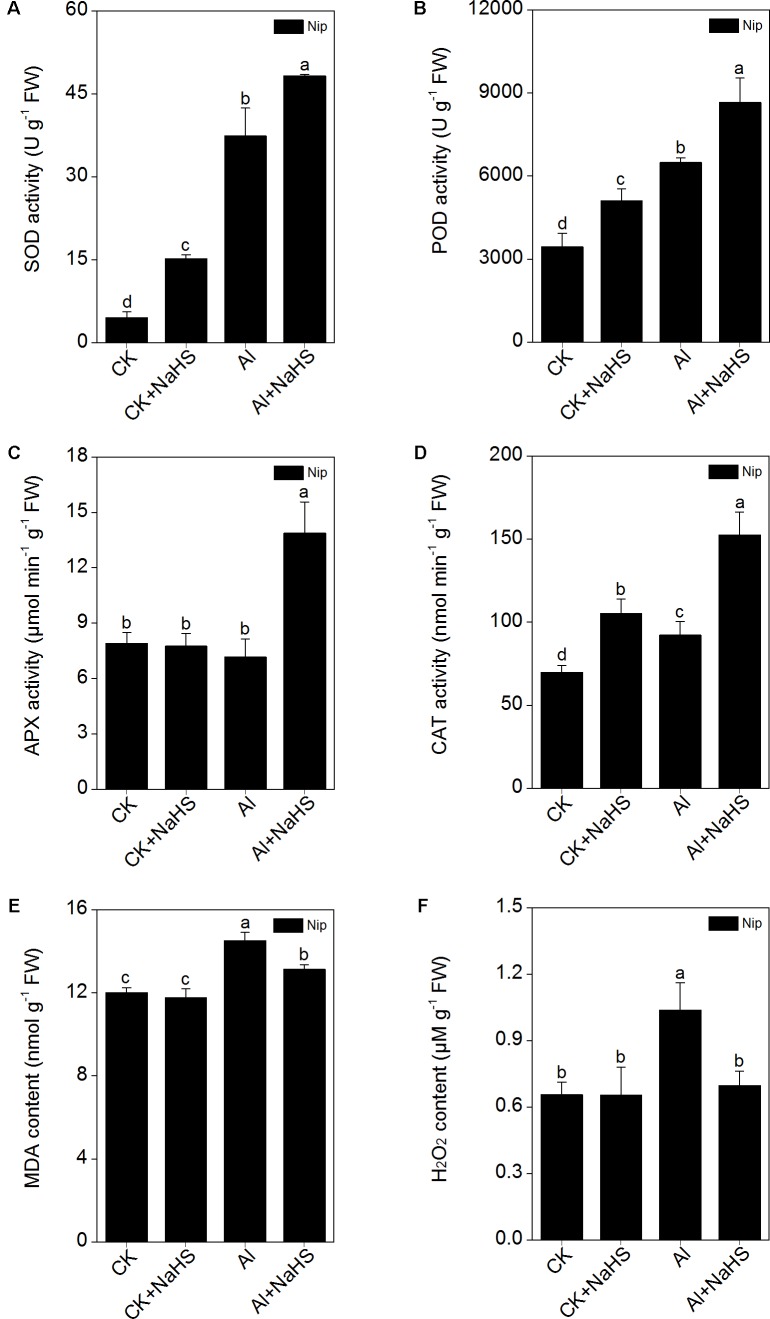
Effect of 2 μM NaHS on SOD **(A)**, POD **(B)**, APX **(C)**, CAT **(D)** activity, MDA **(E)** and H_2_O_2_ **(F)** content in rice roots. Data are means ± SD (*n* = 4). Columns with different letters are significantly different at *P* < 0.05.

The elimination of ROS through antioxidant enzymes is the most common strategy for alleviating Al toxicity (Ezaki et al., 2000; Mittler, 2002). In the present study, the activity of the antioxidant enzymes SOD, CAT, and POD were all significantly increased in response to Al, and further increased by the pretreatment with NaHS under 50 μM Al^3+^ conditions (**Figure 7**). However, the simple application of 50 μM Al^3+^ within 24 h failed to stimulate the activity of APX; only pretreatment with 2 μM NaHS and growth under 50 μM Al^3+^ conditions dramatically increased the activity of APX, indicating that APX may plays an important role in H_2_S-mediated alleviation of Al toxicity via increasing the antioxidative system. In addition, the content of MDA (which can be used to estimate the levels of lipid peroxidation) and H_2_O_2_ in rice roots exhibited similar changes as APX activity (**Figure 7**). These results indicate that H_2_S increases the activities of antioxidant enzymes, likely with APX having a key function, to alleviate Al toxicity in rice.

### H_2_S Modulates the NO Content to Alleviate Al Toxicity

**FIGURE 8:**
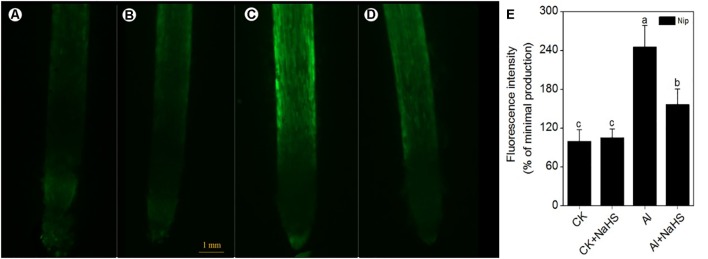
The fluorescence intensity of nitric oxide (NO) under CK **(A)**, CK+NaHS **(B)**, Al **(C)**, Al+NaHS **(D)** conditions, and the relative fluorescence intensity of NO under different treatments **(E)** in rice root tips. The concentration of NaHS is 2 μM, and the concentration of Al is 50 μM. Data are means ± SD (*n* = 10). Scale bar = 1 mm. Columns with different letters are significantly different at *P* < 0.05.

**FIGURE 9:**
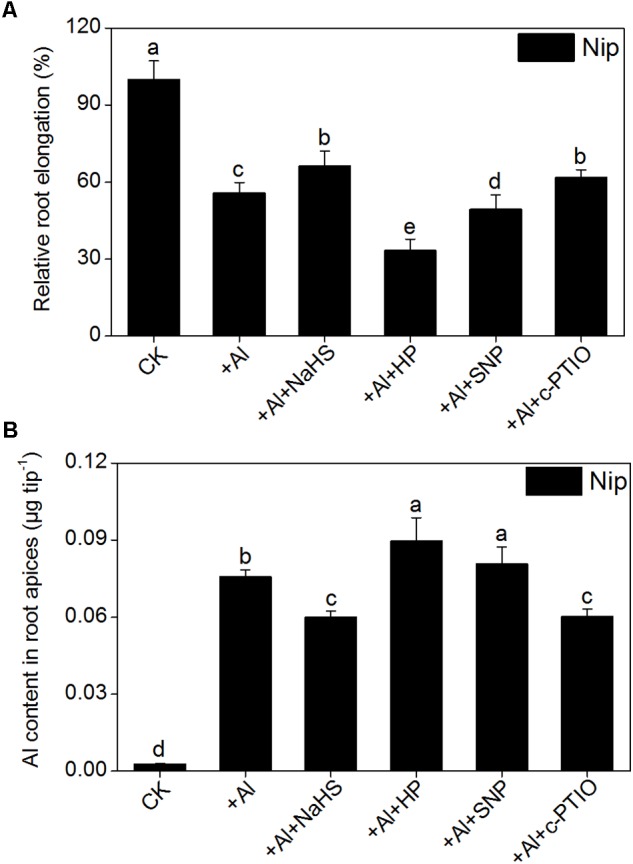
Effects of different treatments on relative root elongation **(A)** and Al content in root apices **(B)**. Data are means ± SD (*n* = 10). Columns with different letters are significantly different at *P* < 0.05.

The signaling molecule nitric oxide (NO) plays a pivotal role in the plant response to Al toxicity, although the results are contradictory. For example, the addition of the NO donor SNP significantly alleviates Al toxicity in red kidney bean (*Phaseolus vulgaris*), wheat (*Triticum aestivum* L.) and *Cassia tora* L (Wang and Yang, 2005; Zhang et al., 2008b; Wang et al., 2010); however, the application of SNP dramatically aggravates Al toxicity in rice bean [*Vigna umbellata* (Thunb.) Ohwi and Ohashi ‘Jiangnan,’ Fabaceae] and rice (*Oryza sativa*) (Zhou et al., 2012; Zhu et al., 2017), and elimination of NO via adding the NO scavenger c-PTIO alleviates Al toxicity in wheat (*Triticum aestivum* L. cv. Yang-5) (Sun et al., 2016). In the present study, the NO content in rice roots significantly decreased in plants pretreated with NaHS compared to the control (**Figure 8**), suggesting that H_2_S might alleviate Al toxicity by decreasing NO content. To explore this notion, the NO donor SNP and the NO scavenger c-PTIO were used to treat rice seedlings under 50 μM Al^3+^ conditions. As shown in **Figure 9**, the addition of SNP significantly decreased root elongation and increased the Al content in root tips under 50 μM Al conditions, whereas c-PTIO had the opposite effect, indicating that NO plays a negative role in the alleviation of Al toxicity. In addition, the presence of the H_2_S scavenger hypotaurine significantly reduced root elongation (**Figure 9**), which was accompanied by an increase in Al contents in roots under high-Al conditions, further confirming the role of H_2_S in alleviating Al toxicity in rice.

## Discussion

In addition to NO and carbon monoxide (CO), H_2_S is another gas that functions as a signaling molecule in plants (Li and Gong, 2011). Accumulating evidence indicates that low doses of H_2_S have a positive effect on seed germination and plant growth, development, and abiotic/biotic stress resistance (Zhang et al., 2009; Yong et al., 2010; Li et al., 2011, 2012; Lisjak et al., 2013). H_2_S has been shown to increase plant resistance to heavy metals. For example, pretreating wheat seeds with NaHS (a H_2_S donor) significantly improves seed germination and seedling growth under Cr, Cu, and Al stress conditions by reducing lipid membrane damage and the deposition of these metals via increasing the activities of amylases, esterases, and antioxidant enzymes (Zhang et al., 2008a, 2010a,b). Pretreating barley seedlings with H_2_S significantly enhances the secretion of citrate, decreases lipid peroxidation and the ROS burst, and increases the amount of the plasma membrane H^+^-ATPase, which in turn improves root growth under Al toxicity conditions (Dawood et al., 2012; Chen et al., 2013). In the current study, the H_2_S content in the rice roots was significantly increased with increasing Al^3+^ content (**Figure 1D**) and 8 h of pretreatment with 2 μM NaHS significantly increased root elongation (**Figures 1G,H**) and decreased Al contents in rice root tips (**Figure 1A**) under 50 μM Al^3+^ conditions. The addition of H_2_S scavenger hypotaurine significantly aggravated the defects in root growth and increased the Al contents in root tips under 50 μM Al^3+^ stress (**Figure 9**), further confirming that H_2_S is involved in alleviation of Al^3+^ toxicity in rice.

Al mainly accumulates in the plant cell wall, and desorption of Al from the cell wall significantly alleviated the Al toxicity for plants. For example, 99.99% of total cellular Al is deposited in the cell wall of the giant alga *Chara corallina* (Rengel and Reid, 1997), and more than 77% of total Al is deposited in the root cell wall in the Al-sensitive wheat cultivar Scout 66 under 10 μM Al^3+^ conditions within 9 h (Ma et al., 2004). Pectin was the first polysaccharide in the cell wall shown to be involved in Al^3+^ tolerance in plants. Chang et al. (1999) demonstrated that nearly 89% of Al in tobacco is present in the cell wall, and approximately 76% is deposited in the pectin fraction (Chang et al., 1999). The difference in Al tolerance between Nipponbare (Al-tolerant cultivar) and Zhefu802 (Al-sensitive cultivar) rice is due to the different pectin contents in the root cell wall and the different levels of methylation in root cell wall pectin (Yang et al., 2008). In Arabidopsis roots, Al is mainly deposited in hemicellulose in the cell wall (Yang et al., 2011), indicating that both pectin and hemicellulose play pivotal roles in mediating Al toxicity in plants. In the current study, rice seedlings pretreated with NaHS had significantly reduced cell wall Al contents due to decreased levels of pectin and hemicellulose in the root cell wall (**Figures 2**, **3**), indicating that H_2_S releases Al bound in the root cell wall by regulating the cell wall polysaccharide content. The enzyme PME catalyzes the demethylation of pectin, leading to the exposure of free carboxylic groups, which have a strong affinity for Al (Blamey and Dowling, 1995; Micheli, 2001); thus, high PME activity leads to a higher Al binding capacity in plant roots, and it has been demonstrated to be negatively associated with Al tolerance in rice (Yang et al., 2013). In the current study, pretreatment with NaHS significantly reduced the activity of PME in rice roots (**Figure 3**), indicating that H_2_S decreases PME activity in roots, thereby decreasing the availability of Al binding sites, which alleviates the inhibition of root elongation under Al-stress conditions.

The expression of *OsSTAR1* and *OsSTAR2* also significantly increased in response to NaHS pretreatment under high Al^3+^ conditions (**Figure 4**). *OsSTAR1* and *OsSTAR2* function in Al tolerance in rice, and the disruption of either gene severely increases Al toxicity. The STAR1-STAR2 complex (encoded by *OsSTAR1* and *OsSTAR2*) functions as an ATP binding cassette (ABC) transporter and transports UDP-glucose from the cytoplasm to the cell wall, resulting in reduced Al deposition, ultimately alleviating Al toxicity (Huang et al., 2009). Pretreatment with H_2_S significantly induced the expression of *OsSTAR1* and *OsSTAR2* under Al^3+^ conditions in the current study (**Figure 4**), further confirming that H_2_S increases Al resistance in rice by reducing Al contents in the cell wall.

Plants often secrete organic acids to alleviate Al toxicity. For example, the addition of Al^3+^ significantly increased oxalic acid efflux from buckwheat. Oxalic acid in the rhizosphere binds with Al^3+^ to form an Al-oxalate complex, which is not phytotoxic to buckwheat (Ma et al., 1997). Citrate acid is the main organic acid present in exudate from rice roots under Al^3+^ conditions, and the secretion of citrate acid is a useful strategy for alleviating Al toxicity in rice (Ishikawa et al., 2000; Ma et al., 2002). *OsFRDL4* encodes a citrate efflux transporter involved in Al-induced secretion of citrate from the roots and is involved in external Al detoxification in rice (Yokosho et al., 2011, 2016). In the present study, the expression of *OsFRDL4* in rice roots was significantly increased under 50 μM Al^3+^ conditions and was further stimulated by pretreatment with H_2_S (**Figure 5**). In addition, pretreatment with H_2_S further increased the citrate acid content in rice root exudates under 50 μM Al^3+^ conditions (**Figure 5**), suggesting that the signaling molecule H_2_S also regulates the secretion of citrate to improve resistance to Al toxicity in rice.

Toxic Al rapidly enters the root cell and inhibits root growth. The trivalent Al ion is transported into root cells by NRAMP ALUMINIUM TRANSPORTER1(NRAT1). In the present study, the expression of *OSNRAT1* significantly decreased in response to NaHS pretreatment, which was accompanied by a decrease in Al levels in cell sap from the root tip (0–1 cm) (**Figure 6**), implying that H_2_S decreases the amount of Al that enters root cells. Once Al enters the cell, the movement of cytoplasmic Al to the vacuole can decrease Al toxicity. OsALS1 is localized to the tonoplast and is a half-size ABC transporter. OsALS1 is responsible for sequestering cytoplasmic Al into the vacuole, and knockout of *OsALS1* leads to an increase in cytosolic and nuclear Al content and increased sensitivity to Al toxicity (Huang et al., 2011). In the current study, the expression of *OsALS1* was significantly induced by Al treatment and was further stimulated by H_2_S pretreatment under Al conditions (**Figure 6**), indicating that H_2_S is also involved in internal Al detoxification by increasing the expression of *OsALS1* in rice roots.

Apart from decreasing the cell wall Al content and compartmentalizing Al in the vacuole, plants also increase antioxidant enzyme activity to resist Al toxicity. The main antioxidant enzymes in plants are SOD, CAT, APX, and POD, all of which are involved in reducing the ROS content (Yamamoto et al., 2002; Kobayashi et al., 2003) and inhibition of lipid peroxidation (Mittler, 2002) in plants under Al-present conditions, thus ameliorating Al toxicity in plants. Dawood et al. (2012) demonstrated that pretreatment with NaHS before Al treatment in barley significantly inhibited the lipid peroxidation via increasing the activity of CAT and POD, thus alleviating the oxidative damage and promoting root growth under Al stress. In the present study, the activities of SOD, POD, and CAT were significantly increased under Al conditions, and pretreatment with NaHS further increased the activities of these three antioxidant enzymes (**Figure 7**), with the activity of APX stimulated only under NaHS pretreatment and Al-present conditions, suggesting that the signaling molecule H_2_S also activates antioxidant systems to reduce the negative effects of Al toxicity on plant growth. As the activity of SOD, POD, and CAT were all similarly increased by NaHS pretreatment under Al-absent conditions, we hypothesize that these three enzymes may be specifically responsive to the NaHS treatment and play an indirect role in detoxification of Al-induced ROS. By contrast, the response of the APX enzyme to NaHS pretreatment only in the presence of Al, accompanied by the similar pattern of change in MDA and H_2_O_2_ contents in roots (**Figure 7**), implies that APX may be the main enzyme responsible for the H_2_S-mediated improved ROS detoxification during Al stress. The APX enzyme is the first line of defense against oxidative stress, using ascorbic acid as specific electron donor to catalyze H_2_O_2_ reduction to water, and is known to be involved in Al detoxication in rice (Sharma and Dubey, 2007). According to a previous study, the content of H_2_O_2_ in rice roots plays an important role in stimulating the synthesis of cell wall polysaccharide, especially in promoting the synthesis and demethylesterification of pectin in rice (Xiong et al., 2015). In the present study, the pectin content and the activity of PME (which is responsible for the pectin demethylesterification) were significantly decreased after pretreatment with NaHS under 50 μM Al^3+^ conditions, just as the activity of APX significantly increased and the H_2_O_2_ content dramatically decreased under NaHS pretreatment and 50 μM Al^3+^ conditions. These corresponding results indicate that the main functions of APX in H_2_S-mediated alleviation of Al toxicity may decrease not only the peroxidation damage from H_2_O_2_, but also pectin synthesis and the activity of PME to diminish the demethylesterification levels via decreasing the H_2_O_2_ content, to ultimately decrease the cell wall Al content in rice roots.

Other signaling molecules might also be involved in alleviating Al toxicity via H_2_S. NO plays a pivotal role in plant responses to Al toxicity and exhibits different effects in different plant cultivars and different culture conditions. The addition of the NO donor SNP significantly enhances the antioxidant capacity to decrease Al toxicity in wheat, soybean and *Cassia tora* L (Wang and Yang, 2005; Zhang et al., 2008b; Wang et al., 2016); however, other studies have demonstrated that NO aggravates Al toxicity to root elongation via regulating cell wall polysaccharose content and structure (Zhou et al., 2012; Sun et al., 2016). For example, when wheat roots were treated with the NO scavenger c-PTIO, PME activity decreased, leading to a higher degree of methylation of pectin in the root cell wall and decreasing the binding capacity between Al and pectin, which in turn promoted root growth and reduced Al levels in roots (Sun et al., 2016). Since both H_2_S and NO have been implicated in the regulation of Al-induced inhibition of root elongation, H_2_S might alleviate Al-induced root inhibition, and subsequently Al toxicity, by modulating NO accumulation. Pretreatment with NaHS significantly decreased the NO content in rice roots under high-Al conditions (**Figure 8**). The addition of the NO donor SNP dramatically increased the inhibitory effect of Al on root elongation and increased the Al content in root tips, whereas the addition of c-PTIO had the opposite effect (**Figure 9**), indicating that the signaling molecule H_2_S reduces NO content to alleviate Al toxicity in rice.

## Conclusion

**FIGURE 10:**
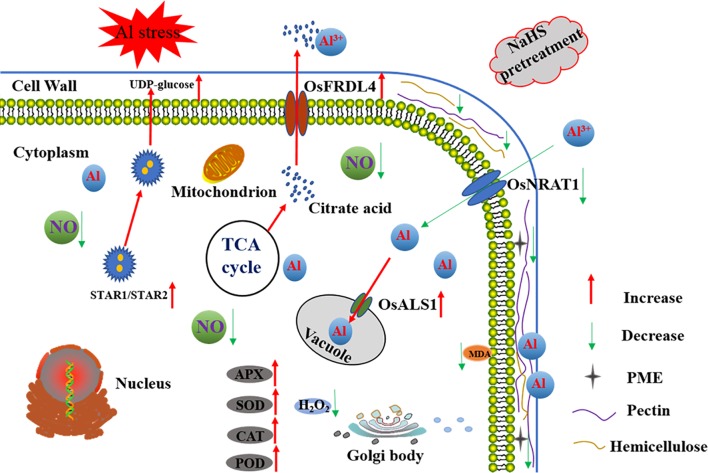
A schematic model for H_2_S-mediated improvement of root elongation in the face of Al toxicity in rice. Pretreatment with NaHS significantly decreased the cell wall Al content via decreasing the cell wall pectin and hemicellulose content and the activity of PME, and increasing the secretion of citrate acid and the expression of *OsSTAR1* and *OsSTAR2*. The pretreatment with NaHS also significantly decreased the translocation of Al from the environment to the cell cytoplasm and promoted its detoxification via increasing the activity of antioxidative enzymes as well as Al transfer from the cytoplasm to the vacuole. The red arrow in the figure denotes increased or enhanced processes, the green arrow in the figure denotes decreased or weakened processes.

This is first report demonstrating that the signaling molecule H_2_S alleviates the inhibition of root growth in rice induced by Al toxicity and decreases Al accumulation in root tips. As show in **Figure 10**, H_2_S functions by decreasing the pectin and hemicellulose contents in the root cell wall, modifying the root cell wall, and decreasing PME activity in roots. Furthermore, H_2_S increases citrate acid secretion from roots, compartmentalizes Al into the vacuole, and increases antioxidant enzyme activity. In addition, H_2_S-mediated alleviation of Al toxicity in rice may involve decreasing the NO accumulation in roots.

## Author Contributions

CQZ, JZ, LS, LZ, and BA performed the research. CQZ analyzed the data and wrote the draft. WH, CZ, ZB, and HS revised the article. CQZ, XC, and QJ designed the research. QJ wrote the article.

## Conflict of Interest Statement

The authors declare that the research was conducted in the absence of any commercial or financial relationships that could be construed as a potential conflict of interest.
